# Unilateral embolization of an arterio-cavernous fistula in the treatment of post-traumatic non-ischemic-priapism: permanent coiling after immediate temporary agent failure

**DOI:** 10.1186/s42155-025-00547-w

**Published:** 2025-04-24

**Authors:** Carolina Dominguez Aleixo, Christoph Erxleben, Emre Baysal, Fabiola Leibling, Maximilian de Bucourt, Bernhard Gebauer, Julian Lenk

**Affiliations:** 1 Department of Radiology, Charité – Universitätsmedizin Berlin, corporate member of Freie Universität Berlin and Humboldt-Universität Zu Berlin, Berlin, Germany; 2 Department of Urology, Charité – Universitätsmedizin Berlin, corporate member of Freie Universität Berlin and Humboldt-Universität Zu Berlin, Berlin, Germany; 3 Department of Radiology, Unfallkrankenhaus, Berlin, Germany

**Keywords:** Angiography, Embolization, Non-ischemic-priapism, Unilateral arterio-cavernous fistula, Penile trauma

## Abstract

A healthy 24-year-old male patient presented with a history of straddle-trauma from a bicycle accident and concomitant non-ischemic-priapism lasting for ten days. On a contrast-enhanced computed tomography scan an arterio-cavernous fistula establishing a connection between the right cavernosal artery and the ipsilateral corpus cavernosum was diagnosed. Super-selective unilateral arterial embolization was performed using gelatin sponge and microcoils. Technical success became evident with the consecutive detumescence of the penis and long-term preservation of baseline urogenital functions.

## Introduction

Penile trauma is a rare emergency, often requiring radiological techniques to accurately classify and assess its extent [1]. Clinically, post-traumatic presentation of penile injuries may involve priapism, characterized by a prolonged erection lacking sexual arousal for its onset, usually due to a disturbance in blood flow [2]. Physiological penile erection involves intact corpora cavernosa and corpus spongiosum, supplied by the penile artery and its three major branches: bulbar, dorsal and cavernosal arteries. Injury to the latter can disturb blood supply to the corpus cavernosum, the anatomical structure responsible for penile volume expansion [3]. Two types of priapism are distinguished: *ischemic* priapism, formerly low-flow or veno-occlusive, is usually idiopathic and characterized by painful blood accumulation in the corpora cavernosa resulting in a localized compartment syndrome [2, 3]; and *non-ischemic* priapism, previously known as high-flow or arterial, often carries a blunt traumatic etiology, as during coitus or straddle injury [4]. Resulting vascular lesions via arterial laceration or damage to the intima with consequent thrombosis may lead to the development of an arterio-cavernous fistula [3, 4]. While the pathophysiology of erectile dysfunction is complex, and several factors may simultaneously play a role (e.g. neurogenic, psychogenic, etc.), vascular injury is a major risk factor for long-term organic erectile dysfunction, especially if untreated [4]. Diagnosis is routinely performed by ultrasound with Color Duplex and Doppler [5]. Severity of erectile dysfunction can be scored according to the International Index of Erectile Function (IIEF), the lower the value, the more severe the condition. An IIEF-value of 22–25 is physiological. Interventional radiological embolization techniques have gained importance in managing post-traumatic vascular pathologies, allowing a safe and effective treatment of arterio-cavernous fistulas [2, 4].

## Case report

**Fig. 1:**
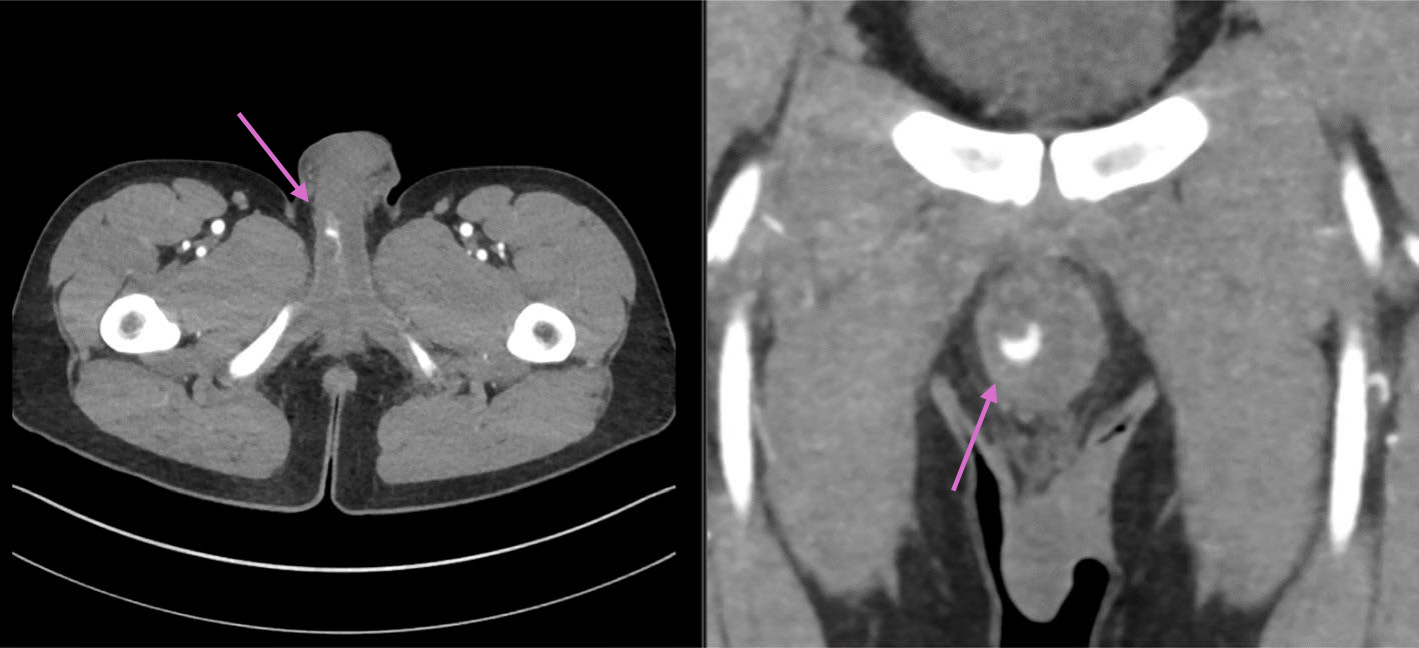
A contrast-enhanced computed tomography (CT) scan of the pelvis displaying a pseudoaneurysm located in the right cavernous body

**Fig. 2:**
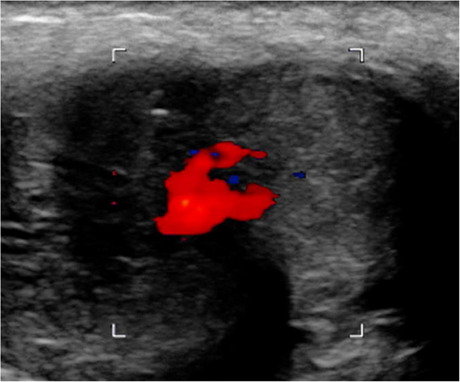
Duplex ultrasound displaying a small correlating pseudoaneurysm in the right cavernous body

A healthy 24-year-old male patient was admitted to a specialized center in a large university hospital with the external diagnosis of priapism and history of straddle-trauma from a bicycle accident. The initial presumptive diagnosis was made by the responsible outpatient urologist. The semirigid, painless penile erection developed post-traumatically within hours and persisted for ten days. A contrast-enhanced computed tomography (CT) pelvic scan displayed a pseudoaneurysm (23 × 5 mm) in the right cavernous body (Fig. 1). Upon admission to the specialized unit, the patient negated priapism-associated pain and disturbances of regular urogenital tasks. No hematoma nor signs of ischemia were noted during physical examination. The patient remained afebrile and in stable general condition. Laboratory results were normal. Similar imaging findings were assessed with Doppler and Duplex ultrasound: a small correlating pseudoaneurysm (Fig. 2) with connection to the ipsilateral cavernosal artery. Insufficient therapeutic results were achieved with local pressure and cryotherapy. After an interdisciplinary case discussion, interventional radiology planned a super-selective embolization of the high-flow system between the artery and the respective cavernous body. Informed consent was obtained, and the intervention was performed one day after transfer from the initial care center.

**Fig. 3:**
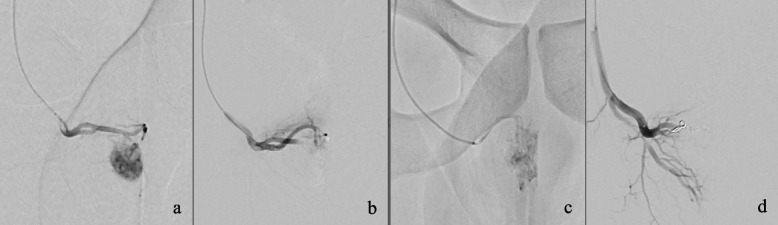
**a** and **b**: arterio-cavernous fistula before and after embolization with the temporary agent **c**: reperfused arterio-cavernous fistula five minutes later **d**: permanent closure of the arterio-cavernous fistula after microcoil embolization

Under local anesthesia, a right femoral retrograde 4 F access of the common femoral artery was established and the right internal iliac artery was catheterized using a 4 F RIM catheter. In the digital subtraction angiography (DSA), the pseudoaneurysm was identified originating from the right internal pudendal artery with fistulous connection to the respective cavernous body. The fistula was selectively catheterized using a 2,4 F microcatheter and initially embolized with a temporary hemostatic agent (CuraSpon®). Complete revascularization was observed five minutes later with incomplete penile detumescence, leading to the decision to occlude the supplying vessel using microcoils (three units, 2 × 4 mm respectively). This resulted in the permanent closure of the fistula and immediate detumescence. Figure 3 displays results before and after closure with the permanent agent. As we attempted to achieve the most distal coil position possible, a “backdoor” closure was not feasible. Finally, the control of the contralateral side did not reveal any further supply of the fistula. Following the intervention, the patient was symptom-free, with no skin changes nor sensory disturbances.

No immediate postoperative complications were noted. The patient was discharged in subjective well-being one day after the procedure, reporting no intercurrences and symptom-free. In a follow-up in the urological outpatient clinic two weeks later, no urinary difficulties, pain nor sensory abnormalities were reported, ejaculatory function was normal, erectile function however unsatisfactory (IIEF-value of 11, equivalent to a moderate erectile dysfunction). The addition of pharmaceutical medication with sildenafil was needed. Three months after the intervention, in the second follow-up in the same institution baseline erectile function was reestablished, the patient had an IIEF of 22. No additional follow-up was needed.

## Discussion

Blunt penile trauma can be caused in a setting of a straddle mechanism, due to impact of the penile shaft against the pelvic bone with the possible formation of a local hematoma. Associated arterial damage may result in the development of an arterio-cavernous fistula [1]. Diagnosis of penile vascular anomalies is routinely performed in an emergency setting with ultrasound with Color Duplex and Doppler [6]. This imaging modality is the gold standard diagnostic tool and additionally plays a role in the follow-up [5]. Treatment of priapism in the context of such post-traumatic vascular anomalies can vary. A first, rather conservative approach consists of the application of local pressure and cryotherapy. Simple watchful waiting is one further option, as non-ischemic priapism may spontaneously resolve [7]. If the condition persists and when treatment is desired in the diagnostic setting of an arterio-cavernous fistula, arterial embolization, as a minimally invasive method for management of posttraumatic non-ischemic priapism, has become the treatment of choice [8]. The decision between the employment of permanent or temporary embolization agents (such as coils or gelatin sponge, respectively) is usually individualized and based on the operator’s experience and expertise. Factors such as the morphology of the vascular abnormality and operator experience should be considered when planning the details of such interventional procedure [9]. Long-term technical success, precision and decreased recurrence are more commonly reported with the application of coils [9]. In cases of parallel psychogenic and/or neurogenic etiologies of erectile dysfunction following blunt penile trauma, oral medication with sildenafil can be useful to satisfactorily restore erectile function [10].

## Conclusion

Our case supports the current literature on arterial embolization being a safe and minimal-invasive treatment option of post-traumatic arterio-cavernous fistulas, as resolution of undesired penile erection was achieved, and baseline erectile function was sufficiently reestablished.

## Data Availability

Data will be made available on reasonable request.
